# TNIK regulation of interferon signaling and endothelial cell response to virus infection

**DOI:** 10.3389/fcvm.2023.1213428

**Published:** 2024-01-09

**Authors:** Khanh M. Chau, Abishai Dominic, Eleanor L. Davis, Sivareddy Kotla, Estefani Turcios Berrios, Arsany Fahim, Ashwin Arunesh, Shengyu Li, Dongyu Zhao, Kaifu Chen, Alan R. Davis, Minh T. H. Nguyen, Yongxing Wang, Scott E. Evans, Guangyu Wang, John P. Cooke, Jun-ichi Abe, David P. Huston, Nhat-Tu Le

**Affiliations:** ^1^ Department of Cardiovascular Sciences, Center for Cardiovascular Sciences, Academic Institute, Houston Methodist Research Institute, Weill Cornell Medical College, Houston, TX, United States; ^2^ Department of Molecular and Cellular Medicine, College of Medicine Texas A&M University, College Station, TX, United States; ^3^ Center for Cell and Gene Therapy, Baylor College of Medicine, College Station, TX, United States; ^4^ Department of Cardiology, The University of Texas MD Anderson Cancer Center, Houston, TX, United States; ^5^ Department of Cellular and Molecular Biology, Baylor College of Medicine, Houston, TX, United States; ^6^ Department of Orthopedic Surgery, Baylor College of Medicine, Houston, TX, United States; ^7^ Department of Pulmonary Medicine, The University of Texas MD Anderson Cancer Center, Houston, TX, United States; ^8^ Department of Microbial Pathogenesis and Immunology, College of Medicine Texas A&M University, College Station, Houston, TX, United States

**Keywords:** TNIK, interferons, STATs, virus infection, RNA-Seq

## Abstract

**Background:**

Traf2 and Nck-interacting kinase (TNIK) is known for its regulatory role in various processes within cancer cells. However, its role within endothelial cells (ECs) has remained relatively unexplored.

**Methods:**

Leveraging RNA-seq data and Ingenuity Pathway Analysis (IPA), we probed the potential impact of TNIK depletion on ECs.

**Results:**

Examination of RNA-seq data uncovered more than 450 Differentially Expressed Genes (DEGs) in TNIK-depleted ECs, displaying a fold change exceeding 2 with a false discovery rate (FDR) below 0.05. IPA analysis unveiled that TNIK depletion leads to the inhibition of the interferon (IFN) pathway [-log (*p*-value) >11], downregulation of IFN-related genes, and inhibition of Hypercytokinemia/Hyperchemokinemia [-log (*p*-value) >8]. The validation process encompassed qRT-PCR to evaluate mRNA expression of crucial IFN-related genes, immunoblotting to gauge STAT1 and STAT2 protein levels, and ELISA for the quantification of IFN and cytokine secretion in siTNIK-depleted ECs. These assessments consistently revealed substantial reductions upon TNIK depletion. When transducing HUVECs with replication incompetent E1-E4 deleted adenovirus expressing green fluorescent protein (Ad-GFP), it was demonstrated that TNIK depletion did not affect the uptake of Ad-GFP. Nonetheless, TNIK depletion induced cytopathic effects (CPE) in ECs transduced with wild-type human adenovirus serotype 5 (Ad-WT).

**Summary:**

Our findings suggest that TNIK plays a crucial role in regulating the EC response to virus infections through modulation of the IFN pathway.

## 1 Introduction

TNIK, a serine and threonine protein kinase, was initially characterized by Fu et al. in 1999 (1). It belongs to the germinal center kinases (GCKs), a subgroup of the Sterile 20 (STE20) family of MAP kinase kinase kinase kinases (MAP4K) (1). TNIK likely functions as an upstream regulator of c-Jun N-terminal kinase (JNK), a member of the mitogen-activated protein kinase (MAPK) family, through its C-terminal regulatory domain, similar to other GCK family members. However, the precise regulatory mechanisms are not yet fully understood (1). Its role in regulating other MAPK family members, such as p38 and ERK, remains unclear (1, 2). While Fu et al. did not propose direct regulation of NF-*κ*B by TNIK (1), Shkoda et al. later reported the association of TNIK with TRAF6/TAB2 and its activation of the NF-*κ*B pathway in Epstein-Barr virus (EBV)-transformed B cells, independently of its kinase activity (3). In addition to its MAP4K-related functions, TNIK is involved in actin cytoskeleton regulation through the phosphorylation of gelsolin, which leads to F-actin fragmentation and capping (1). TNIK also acts as a downstream effector and interaction partner of Rap2 (but not Rap1), a small GTP-binding protein in the Ras family, in regulating the actin cytoskeleton (2). Nevertheless, further studies are required to elucidate the specific physiological stimuli that activate TNIK in F-actin regulation.

The focus on TNIK shifted significantly when Mahmoudi et al. reported its direct binding and regulation of the *β*-catenin and T-cell factor-4 (TCF-4) transcriptional complex (4, 5). This complex plays a crucial role in activating Wnt signaling-induced proliferation in colorectal cancer and leukemia stem cells, with TNIK also responsible for phosphorylating TCF4/TCF7L2 (transcription factor-7 like 2) (4–9). Consequently, most research efforts have been concentrated on understanding the involvement of TNIK in cancer growth, leading to the development and testing of small-molecule TNIK inhibitors like NCB-0846 for their anti-tumor effects (4, 10, 11). While TNIK is highly expressed in the brain and enriched in the postsynaptic density of glutamatergic synapses (12–16), its role in the brain remains incompletely understood. TNIK is known to regulate actin dynamics, cell morphology, and neuronal structure (2, 17, 18) and also interacts with the multifunctional scaffold protein Disrupted In Schizophrenia 1 (DISC1) (19, 20), which is associated with psychiatric disorders (19–21). Transcriptional profiling, genome-wide association studies, and functional genomic network analysis suggest that TNIK might function as a psychiatric risk gene (12, 15, 22, 23). *Tnik* knock-out mice display hyper-locomotor behavior, which can be reversed by GSK3β inhibitors (24). However, the role of TNIK in ECs remains largely unexplored (25, 26). Our RNA-seq analysis, combined with IPA, has revealed that TNIK plays a crucial role in regulating the IFN signaling pathway and modulating cytokines and chemokines in ECs. Furthermore, our experiments involving EC transduction with adenovirus have demonstrated the importance of TNIK in protecting ECs against cytopathic effects (CPE) induced by Ad-WT.

## 2 Materials and methods

### 2.1 Antibodies and reagents

We obtained antibodies for STAT1 (#14994), STAT2 (#72604), TNIK (#32712), and *β*-actin (#4970) from Cell Signaling (Danvers, MA). The MX1 (#sc-271024) antibody was acquired from Santa Cruz Biotechnology (Dallas, TX). Sigma-Aldrich (St. Louis, MO) provided the following reagents: Protease inhibitor cocktail (#P8340), PMSF (#36978), NEM (#E3876), Diphenyleneiodonium chloride (D2926), and FLAG tag antibody (#F3165). The Lipofectamine 2000 transfection reagent (#11668027) was purchased from Thermo Fisher Scientific (Waltham, MA).

### 2.2 Cells

HUVECs were obtained through collagenase digestion of the endothelium of human umbilical cord veins and cultured on gelatin type A-coated dishes/flasks (0.2%; #901771; MP Biomedicals, Santa Ana, CA, USA) in EC medium [ECM, #1001, Science Cell, San Diego, CA, USA]. This study was approved by the Houston Methodist Research Institute (HMRI) Institutional Review Board (IRB Pro00020559), and informed consent was not required. HAECs were generously provided by Dr. Aldons J. Lusis (UCLA, David Geffen School of Medicine).

### 2.3 Transfection

We used a siRNA targeting the nucleotide coding sequence 843–857 of human TNIK (siTNIK: 5’- GGACCCUUCUCAGAAGUUCCCUCAA) obtained from Sigma (Burlington, MA, USA). Additionally, a non-target control sequence (siCTRL) was procured from Thermo Scientific. The transfection of siRNA and plasmids was conducted as previously described (27, 28) in GIBCO Opti-MEM reduced serum medium (#31985070; Thermo Fisher Scientific) containing Plus reagent (#11514015) and Lipofectamine (#18324020) acquired from Life Technologies. Cells were allowed to recover for 48 h after siRNA transfection or 24 h after plasmid transduction before further processing.

### 2.4 RNA-Seq

Total RNA was extracted from HAECs after 48 h of transfection with siCTRL or siTNIK (50 nM) using the RNeasy Plus Micro Kit (#74034, QIAGEN). The RNA samples were sent to the Beijing Genomic Institution (BGI, Shenzhen, China) for mRNA preparation, library construction, and sequencing utilizing a BGISEQ-500. Clean tags were aligned to reference genomes and genes sourced from the Mice Genome Annotation Project, permitting up to one mismatch. All paired-end RNA-seq reads were aligned to the human genome (gencode HG38) using Kallisto (v0.46.0) with default parameters. Gene expression level and significance of DEG were computed by DESeq2 (v2.0.12). Gene expression was quantified as transcripts per million (TPM). DEGs were defined by DESeq2 with a *Q* value threshold of ≤0.05. Transcriptional profiles and heatmaps of siCTRL- and siTNIK-transfected cells were generated using MORPHEUS https://software.broadinstitute.org/morpheus/. We conducted pathway-enriched analysis for curated gene sets categorized by biological functions and diseases using DAVID (https://david-d.ncifcrf.gov). Each gene ontology term was assigned a *p*-value using a modified Fisher's exact test. The biological functions and diseases were based on curated findings in the literature stored in the IPA.

### 2.5 qRT-PCR

Total RNA was extracted from HUVECs after 48 h of transfection with siCTRL or siTNIK (50 nM) using the RNeasy Plus Micro Kit (#74034, QIAGEN). cDNAs were synthesized with iScript Reverse Transcription Supermix for RT-qPCR (Cat#1708841, Bio-Rad Laboratories, USA), following the manufacturer's protocol. Mixtures for qRT-PCR reactions included cDNA and 0.5 µM of both forward and reverse primers. The qRT-PCR was initiated at 95°C for 3 min, followed by 40 cycles of denaturation at 95°C for 10 s and annealing at 55°C for 30 s. QuantStudio Real-Time PCR system from Applied Biosystems was used for qRT-PCR data acquisition. The comparative C_t_(^2−ΔΔCt^) method was employed to quantify changes in mRNA expression relatively. In this method, cycle threshold (Ct) values of target genes were normalized to those of reference genes (29). All qRT-PCR primers were sourced from Sigma-Aldrich, and the primer sequences can be found in Supplementary Material Table S1.

### 2.6 Immunoblotting

Protein extraction was performed in RIPA buffer (#20-118 EMD Millipore, Billerica MA, USA) containing 50 mM Tris-HCl (pH 7.4), 150 mM NaCl, 1 mM ethylenediaminetetraacetic acid, 1% Nonidet *P*-40, 0.1% sodium dodecyl sulfate, and 0.25% sodium deoxycholate. The buffer was supplemented with mammalian protease inhibitor cocktail (#P8340; Sigma-Roche, Mannheim, Germany), 1 mM phenylmethylsulfonyl fluoride (#36978; Thermo Fisher Scientific), and 10 mM N-ethylmaleimide (#E3876; Sigma-Roche, Mannheim, Germany) (28). After centrifugation at 15,000 rpm for 20 min at 4°C to remove debris, protein concentration was determined using the DC™ Protein Assay Kit I (#5000111; Bio-Rad, Hercules CA, USA). Equal protein amounts in SDS-gel loading buffer were loaded onto SDS-PAGE gels and then electrotransferred onto Immobilon-*P* transfer membranes (#IPVH00010; Merck Millipore, Burlington, MA). The membranes were incubated in 3% BSA/TBST (10 mM Tris-HCl, 0.15 M NaCl, 0.1% Tween 20, pH 8.0) at room temperature for 1 h, washed in TBST, and incubated with each specific antibody (diluted to 1:500–1:1,000) with mild agitation overnight at 4°C. After washing three times for 10 min each in TBST, the membranes were incubated with HRP-conjugated secondary antibodies (diluted to 1:10,000–1:20,000), washed three times for 10 min each in TBST, and chemiluminescence was detected using ECL substrate (#NEL105001EA; Perkin Elmer, Waltham, MA, USA). Signal intensities from immunoblotted membranes were quantified by densitometry using ImageJ.

### 2.7 Cytokine assay

To detect secreted cytokines, we utilized the Proteome profiler array (#ARY005B, R&D systems, Minneapolis, MN) and followed the manufacturer’s instructions to perform ELISA.

### 2.8 Virus transduction

We employed a first-generation human type 5 adenovirus expressing GFP (Ad-GFP), which is replication incompetent due to E1-E4 deletion, to assess virus transduction efficiency and determine the multiplicity of infection (MOI) (30). GFP expression was validated by transducing HUVECs with Ad-GFP and subsequently confirming GFP expression 24 h post-transduction using a Keyence BZ-X810 fluorescence microscope at 20× magnification. In experiments involving wild-type adenovirus, HUVECs were transduced with replication-competent human type 5 adenovirus at an MOI of 60. The ratio of virus particle (vp) to a plaque-forming unit (pfu), denoted as vp/pfu, ranged from 23 to 42.

### 2.9 Viral plaque assay

HUVECs were transfected with either siCTRL or siTNIK (50 nM). Following a 48-hour incubation, these cells were transduced with Ad-WT at an MOI of 60. We closely monitored the CPE daily through microscopic examination. Cells exhibiting CPE were identified based on three primary morphological alterations, which include (1) Changes in cell morphology, resulting in a round shape; (2) Fusion with adjacent cells to form syncytia; and (3) The presence of cytoplasmic/nuclear vacuoles due to the accumulation of viral components (31–35). The percentage of cells exhibiting CPE after Ad-WT transduction was quantified by calculating the ratio of cells with CPE to the total cell count. To determine the quantity of infectious virus particles within these cells, a series of three freeze-thaw cycles were performed at the 72-hour post-viral transduction time point. Subsequently, a plaque-based assay was conducted using an immunohistochemical approach to measure the number of infectious viral particles within HEK293 cell lysates (36).

### 2.10 Flow cytometry

To assess virus transduction efficiency, HUVECs were initially transfected with siCTRL or siTNIK (50 nM). After 48 h, they were transduced with Ad-GFP at an MOI of 60. Subsequently, these cells were harvested through trypsinization, followed by a PBS wash and resuspension in 5% BSA in PBS. The percentage of cells expressing GFP was quantified using the BD Accuri™ C6 Plus Flow Cytometer (BD Biosciences). Data analysis was conducted using FlowJo (Version 10; Treestar US, Ashland, OR).

### 2.11 Statistical analysis

To compare differences among more than two independent groups, we employed a one-way analysis of variance (ANOVA) and subsequently conducted Bonferroni *post hoc* tests for multiple group comparisons using GraphPad Prism (GraphPad Software, San Diego, CA, USA). Significance levels were denoted in the figures as follows: one asterisk (*) for *p*-value less than 0.05, two asterisks (**) for *p-*values less than 0.01, and three asterisks (***) for *p-*values less than 0.001.

### 2.12 Data availability

The RNA-seq data has been deposited in the NCBI's Gene Expression Omnibus database under the Accession number GSE236529 with the following token: wtchyemijfcdjad. For any additional data, analytic methods, or study materials that are essential for supporting the findings of this study, please feel free to request them from the corresponding author, and they will be made available upon reasonable request.

## 3 Results

### 3.1 Assessing the functional impact of TNIK depletion in ECs using IPA

**Figure 1:**
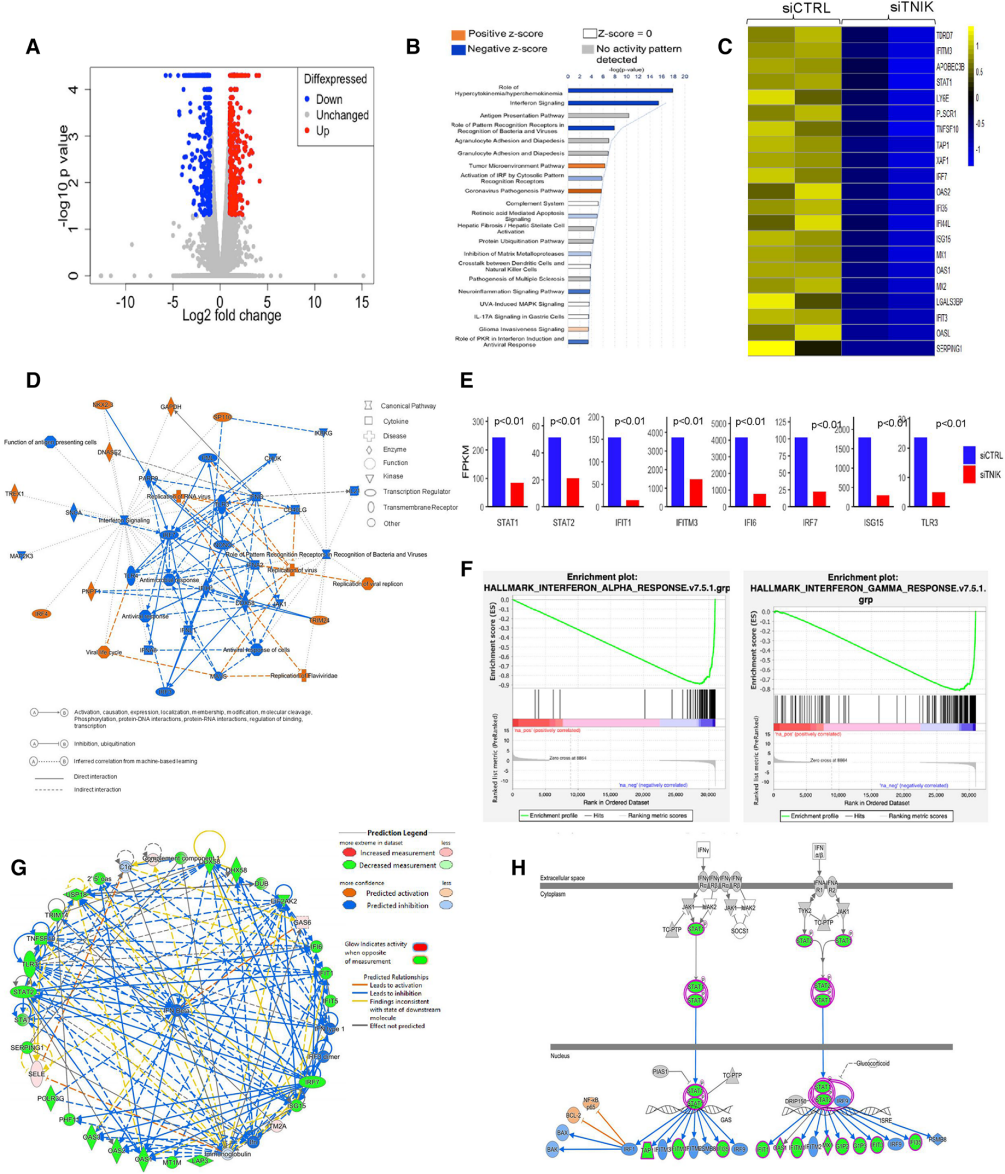
IPA analysis demonstrates downregulation of IFN networks due to TNIK depletion: (**A**) volcano plot depicting DEGs in siTNIK-transfected HAECs vs. siCTRL-transfected HAECs, where “Red” dots indicate upregulated DEGs and “Blue” dots indicate downregulated DEGs. (**B**) IPA-based Pathway Analysis identifies key pathways affected due to TNIK depletion. The color-coded scale represents activation *z*-score values, where “Orange” indicates upregulation, “Blue” indicates downregulation, “White” indicates a *z*-score of 0, and “Gray” indicates data not available. The top two significantly downregulated pathways due to TNIK depletion are the “Role of Hypercytokinemia/Hyperchemokinemia” and the “IFN signaling pathway”. (**C**) A list of 21 significantly downregulated IFN-related genes due to TNIK depletion, indicates the role of TNIK in regulating inflammation via the IFN pathway in ECs. (**D**) An IPA-generated Graphical Summary illustrates a reduction in the IFN pathway and Antimicrobial/Antiviral responses as major affected pathways after TNIK depletion. The Network's Organization and Functional Linkages are determined by the default settings of IPA (protein fold-change cut-off values of >1.33 for activation and <−1.33 for inhibition). In this representation, IPA selects and connects a subset of the most statistically significant entries related to TNIK depletion, including canonical pathways, upstream regulators, diseases, and biological functions, providing a concise overview of the biological activities linked to TNIK depletion. In the color code, “Orange” indicates predicted activation, and “Blue” represents predicted inhibition. (**E**) Comparison of the expression levels (by FPKM values) of genes relating to IFN pathway in siTNIK- and siCTRL- transfected HAECs. Several key IFN Gene Set Pathways are downregulated (*p* < 0.01). (**F**) GSEA reveals downregulation of both IFN-α and IFN-β responses in ECs due to TNIK depletion. (**G**) IPA Network Analysis showing downregulation of several IFN genes with predicted downregulation of IFN-β responses due to TNIK depletion. (**H**) IPA Pathway Prediction Analysis demonstrates that TNIK depletion leads to downregulation of STAT1 and STAT2, resulting in subsequent downregulation of multiple downstream IFN genes.

To assess the functional consequences of TNIK depletion within ECs, we transfected HAECs with either siCTRL or siTNIK. 48 h after transfection completion, we extracted total RNA from the cells and conducted RNA-seq. We identified a group of genes in the siTNIK-transfected HAECs with significant DEGs (log2 fold change >1, *p-*value <0.05). Subsequently, we conducted a core analysis using the knowledge-based IPA database (QIAGEN, application build 377306M [2016-03-16] and content version 27216297 [2016-03-16]). Our analysis identified 312 upregulated DEGs and 355 downregulated DEGs in siTNIK-transfected HAECs (Figure 1A, and Supplementary Material Figure S1A).

Our analysis identified the top three most significant categories associated with TNIK depletion, including: (1) Role of Hypercytokinemia/Hyperchemokinemia [-log(*p-*value) 17.9, *z*-score −4.359, *p-*value 1.21E-18]; (2) IFN signaling [-log(*p-*value) 15.5, *z*-score −3.606, *p-*value 2.85E-16]; and (3) Role of Pattern Recognition Receptors in Recognition of Bacteria and Viruses [-log (*p-*value) 7.91, *z*-score −2.828, *p-*value 1.24E-08]. Moreover, curated gene sets associated with the Role of Hypercytokinemia/hyperchemokinemia and the IFN signaling are predicted to be downregulated as a consequence of TNIK depletion (Figure 1B, Supplementary Material Table S2).

The predicted biological impact of TNIK depletion on gene expression is visually depicted through heat maps (Supplementary Material Figure S1B). The analysis reveals a significant upregulation of gene sets related to infectious diseases, including those linked to virus infection (*z*-score 3.452, *p-*value 3.62E-27), virus replication (*z*-score 3.801, *p-*value 4.1E-24), and virus production (*z*-score 3.011, *p-*value 9.29E-06). In contrast, gene sets associated with the inflammatory response category exhibit significant downregulation, encompassing genes associated with antiviral responses (*z*-score −3.518, *p-*value 8.38E-38), antimicrobial responses (*z*-score −3.518, *p-*value 2.09E-28), cellular immune responses (*z*-score −2.727, *p-*value 6.87E-11), and innate immune responses (*z*-score −1.112, *p*-value 5.87E-18) (Supplementary Material Figures S1B,C and Supplementary Material Tables S3,S4).

We found that gene sets related to the immune cell trafficking category, specifically cellular infiltration by phagocytes (*z*-score −2.411, *p-*value 2.02E-07, Supplementary Material Table S5), were predicted to be downregulated as a result of TNIK depletion. Additionally, gene sets associated with the cellular movement category, such as cell movement of myeloid cells (*z*-score −2.295, *p-*value 1.03E-10, Supplementary Material Table S6), as well as those in the organismal injury and abnormalities category, like hematologic cancer of cells (*z*-score 2.064, *p-*value 4.08E-11, Supplementary Material Table S7); hematological diseases category, such as lymphatic neoplasia (*z*-score 2.064, *p-*value 8.81E-11, Supplementary Material Table S8); and the cancer category, such as neoplasia of cells (*z*-score 2.121, *p-*value 5.19E-14, Supplementary Material Table S9), are also expected to be influenced as a result of TNIK depletion.

Our gene enrichment analysis, taking into consideration both directional gene expression changes and supported causal relationships from the literature, indicates that genes associated with viral replication, replication of viral replicon, viral life cycle, replication of RNA virus, and replication of Flarividae are upregulated in siTNIK-transfected HAECs in comparison to siCTRL-transfected HAECs (*z*-score 3.452, Figure 1D). Moreover, among the 28 genes linked to the IFN pathways and Role of Hypercytokinemia/hyperchemokinemia, their directional changes align with an increase in viral infection (Supplementary Material Table S2).

### 3.2 TNIK depletion downregulates IFN signaling in ECs

In our study, we observed that HAECs transfected with siTNIK showed the downregulation of 21 out of 29 IFN-related genes, as identified by Gene Set Enrichment Analysis (GSEA) when compared to siCTRL-transfected HAECs (Figure 1C). These results suggest that TNIK likely has a regulatory role in IFN signaling within ECs. We further examined the expression of genes [by Fragments Per Kilobase if transcript per Million mapped reads (FPKM) values] related to IFN signaling pathway, including STAT1, STAT2, IFIT, IFIT3, and IFI6, using gene tracks from the Bigwig files in the Integrative Genomics Viewer (IGV). Our analysis revealed a downregulation in the expression of these genes (Figure 1E).

To validate the inhibition of IFN signaling resulting from TNIK depletion, we analyzed an IPA-generated graphical summary. This graphical summary, shown in Figure 1D, highlights the impact of TNIK depletion on various biological activities and signaling pathways. The protein network supports the idea that depleting TNIK hampers IFN signaling and the antiviral response while promoting viral replication and infection. This visual representation is based on an algorithm and machine learning techniques that establish connections among significant entries related to TNIK depletion, including canonical pathways, upstream regulators, diseases, and biological functions. It provides a clear depiction of the relationship between TNIK and IFN signaling, shedding light on connections that may not have been previously apparent in the QIAGEN Knowledge Graph. The figure underscores the inhibitory effect of TNIK depletion on IFN signaling and the antiviral response while enhancing viral replication and infection.

We performed GSEA to analyze both IFN-α/β and IFN-γ responses in siTNIK-transfected HAECs compared to siCTRL-transfected HAECs. In both scenarios, we observed negative normalized enrichment scores of −2.25 and −2.21, respectively. These scores indicate that TNIK depletion leads to the downregulation of IFN responses in ECs (Figure 1F). Moreover, IPA network analysis highlighted the IFN-α/β network as the most significantly enriched network affected by TNIK depletion. Using a comprehensive and meticulously curated database of molecular interactions and functional annotations, we established molecular and functional connections between IFN-α/β (the central molecule) and genes influenced by TNIK depletion. Among the 34 identified genes, 25 (74%) exhibited downregulation, 4 (12%) remained unaffected, and 5 (15%) displayed upregulation due to TNIK depletion (Figure 1G). In addition to its role in the IFN response, TNIK may also regulate the expression of two key transcription factors within the IFN signaling pathway, STAT1 and STAT2 (Figure 1H).

**Figure 2:**
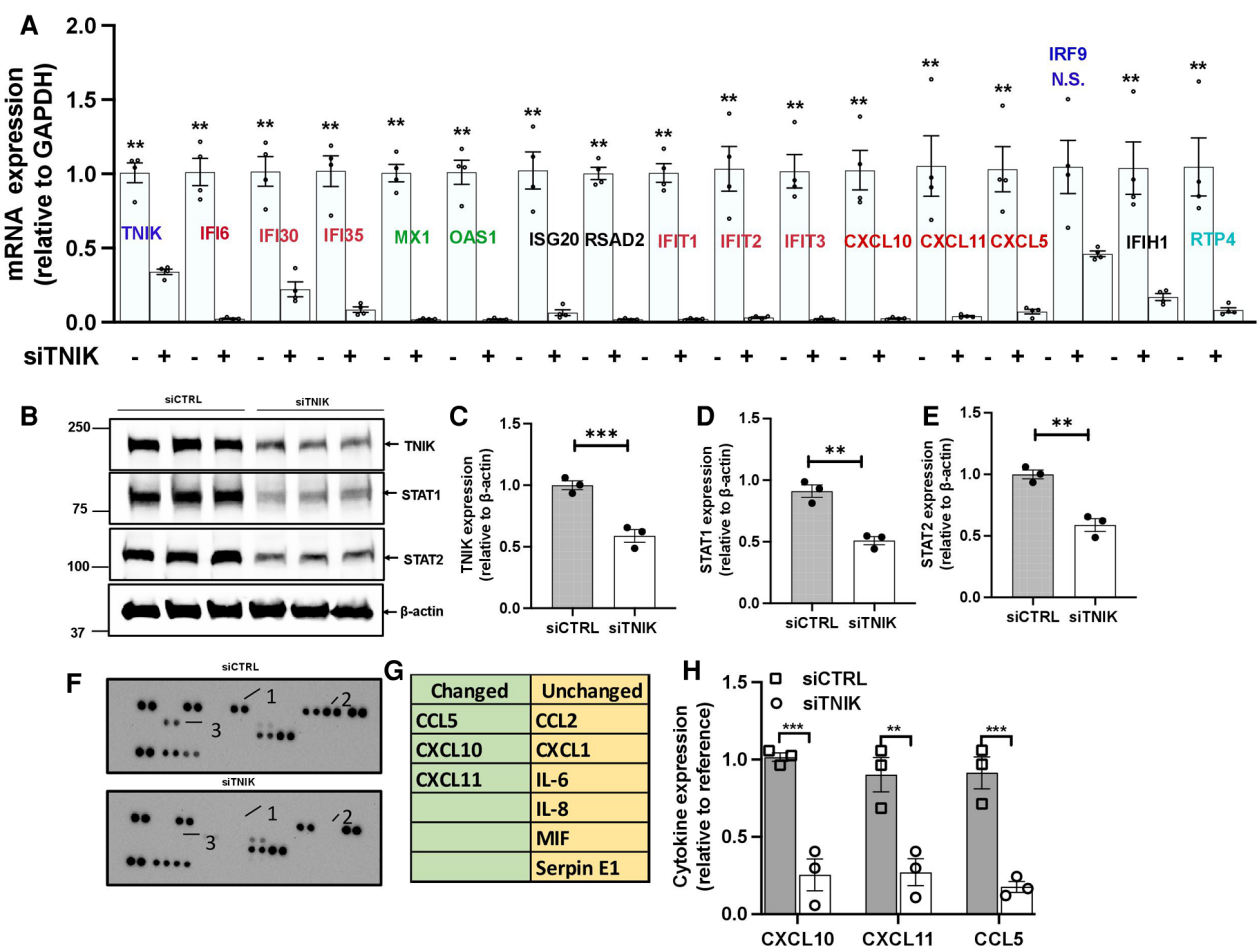
TNIK modulation of STAT-mediated IFN signaling. (**A**) qRT-PCR analysis demonstrates downregulated mRNA expression of *Tnik* and IFN-related genes as a result of TNIK depletion. The graph shows mean ± SEM (*n* = 4, ** indicates *p *< 0.01). (**B**) Immunoblotting results show the expression of TNIK, STAT1, STAT2, and actin following TNIK depletion. (**C,D,E**) The graphs illustrate the fold change in TNIK, STAT1, and STAT2 expression relative to actin (used as a control) due to TNIK depletion. Mean ± SEM (*n* = 3, ** indicates *p* < 0.01). (**F,G**) ELISA assay reveals secreted cytokines in the conditioned medium of siTNIK- vs siCTRL-transfected HUVECs. (**H**) The graphs display cytokine expression relative to a reference. Mean ± SEM (*n* = 3, ** indicates *p* < 0.01, *** indicates *p *< 0.001).

To validate our key findings from the RNA-seq data analysis, we conducted qRT-PCR using HUVECs transfected with either siCTRL or siTNIK. We measured the relative changes in the mRNA expression level of IFN signaling-related genes, including *ifi6, ifi30, ifi35, isg20, ifit1, ifit2, ifit3, irf9, ifih1,* and *rtp4.* 48 h after siTNIK transfection, we observed a reduction in the mRNA expression level of TNIK, which, in turn, led to decreased mRNA expression levels of all IFN signaling-related genes, except for *irf9* (Figure 2A). This aligns with the established role of IRF9 as a negative regulator of the IFN pathway (37).

We also observed a reduction in the mRNA expression levels of crucial genes involved in viral replication, including IFN-induced GTP binding protein Myxovirus resistance protein 1 (*mx1*), 2-prime,5-prime oligoadenylate synthetases 1 (*oas1*), and radical S-adenosyl methionine domain-containing protein 2 (*rsad2*), in siTNIK-transfected cells (Figure 2A). TNIK depletion in ECs resulted in decreased mRNA expression levels of key cytokines, specifically C-C motif chemokine ligand 5 (*ccl5*), chemokine C-X-C motif ligand 10 (*cxcl10*), and *cxcl11* (Figure 2A)*.* These cytokines play a crucial role in recruiting adaptive immune cells. Moreover, we observed a decrease in the protein expression levels of STAT1 and STAT2 in siTNIK-transfected ECs (Figures 2B–E), providing further evidence supporting the role of TNIK on IFN signaling-related expression.

To validate the effects of TNIK depletion on cytokine secretion, we analyzed cytokines secreted by siTNIK-transfected HUVECs using a cytokine array. In the conditioned medium, we assessed the secretion of CCL2, CXCL1, IL6, IL8, MIF, Serpin E1, CCL5, CXCL10, and CXCL11. Our analysis revealed that the secretion of CCL5, CXCL10, and CXCL11 was undetectable in the conditioned medium from siTNIK-transfected HUVECs, unlike that from siCTRL transfected cells (Figures 2F–H). The results obtained through qRT-PCR, immunoblotting, and cytokine array collectively corroborate the anticipated functional impact of TNIK depletion in ECs, which involves the downregulation of IFN signaling and cytokine secretion.

### 3.3 Increased susceptibility to virus infection upon TNIK depletion

IFNs are naturally produced by cells in response to various stimuli, including virus infections. Activation of the IFN signaling pathway leads to the induction of cellular responses, including the upregulation of genes involved in antiviral defense, immune modulation, and cell growth (38, 39). The downregulation of IFN signaling resulting from TNIK depletion may ultimately increase susceptibility to virus infection. To investigate this possibility, we employed adenoviral infection, a known activator of IFN signaling (40–42).

**Figure 3:**
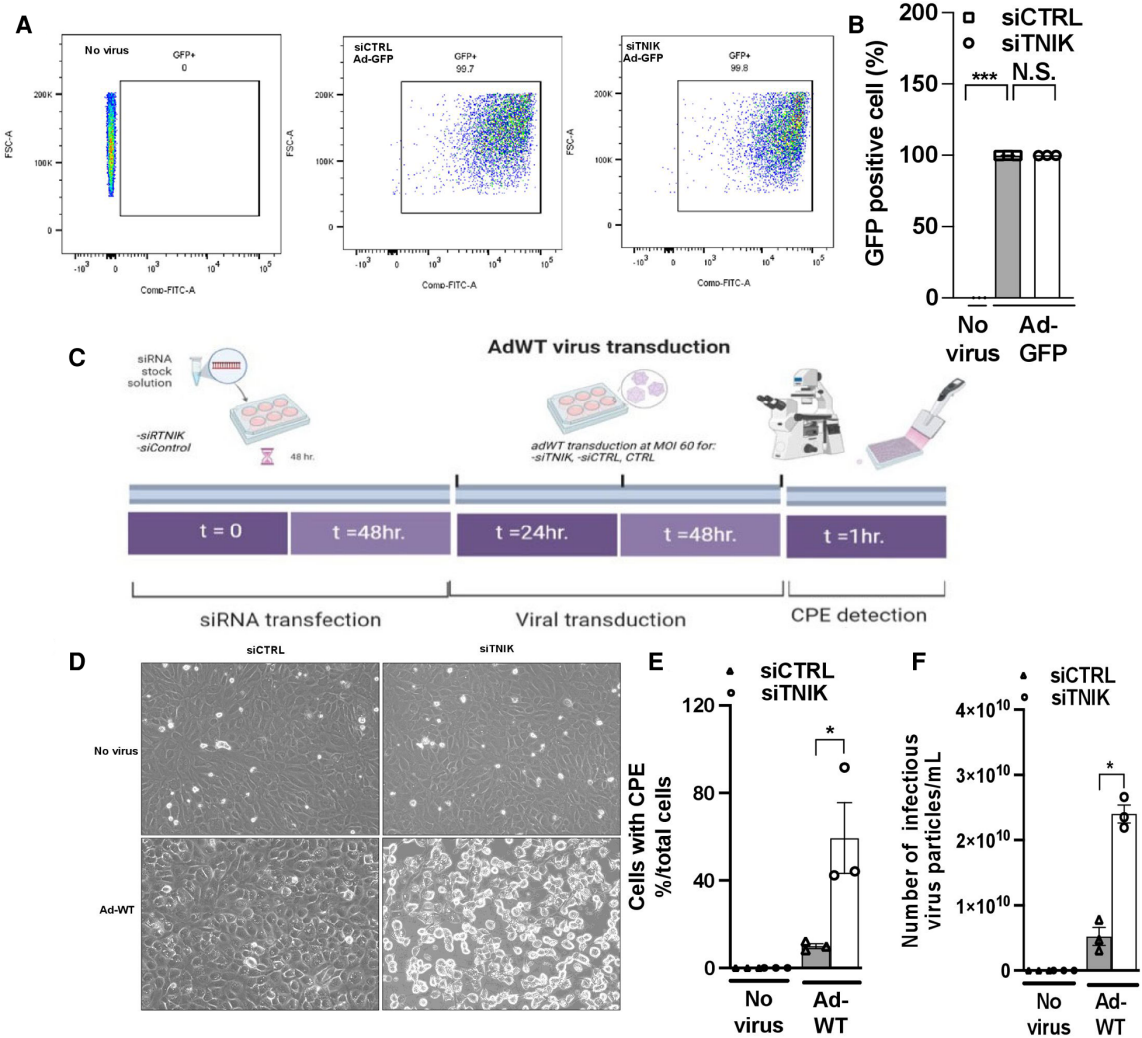
TNIK depletion increases susceptibility to virus infection. (**A**) HUVECs were initially transfected with siCTRL or siTNIK (50 nM). After a 48-hour incubation, these cells were transduced with Ad-GFP (MOI 60). The percentage of GFP-expressing cells in siTNIK and siCTRL-transfected HUVECs was quantified 24 h after Ad-GFP transduction using flow cytometry. (**B**) Quantification of GFP-expressing cells 24 h after Ad-GFP transduction, presented as the mean ± SEM (*n* = 3, *** indicates *p* < 0.001, N.S. not significant). (**C**) Experimental design for assessing the role of TNIK in ECs using an Ad-WT model: HUVECs were categorized into three groups- those without siRNA transfection (CTRL), those transfected with siTNIK, and those transfected with siCTRL. The cells were first transfected with siCTRL or siTNIK (50 nM). After a 48-hour incubation, these cells were transduced with Ad-WT (MOI 60). Cell cultures were observed daily for the development of cytopathic effects (CPE) induced by Ad-WT under a microscope until CPE was detected, which occurred 72 h after Ad-WT transduction. (**D**) Cells exhibiting CPE were observed in siTNIK-transfected cells after 72 h of Ad-WT transduction, while other cells maintained an intact monolayer (scale bar: 20 µm). (**E**) The percentage of cells exhibiting CPE after Ad-WT transduction was quantified by calculating the ratio of cells with CPE to the total cell count. The graph shows mean ± SEM (*n* = 3, * indicates *p* < 0.05). (**F**) A plaque-based assay to quantify infectious virus particles in siTNIK-transfected HUVECs compared to those in siCTRL-transfected HUVECs. The graph shows mean ± SEM (*n* = 3, * indicates *p* < 0.05).

Initially, we transduced HUVECs with Ad-GFP at 5 × 10^12^ particles/ml, 1.2 × 10^11^ plaque-forming unit (pfu)/ml at various MOI values ranging from 60 to 475. Successful infection was confirmed by GFP expression 24 h after transduction at an MOI of 60 (Supplementary Material Data Figure S2). Subsequently, we transduced both siCTRL and siTNIK-transfected HUVECs with Ad-GFP (MOI 60) and quantified the percentage of GFP-expressing cells using flow cytometry. Our analysis revealed that approximately 99% of cells expressed GFP, with no significant difference between the two groups (Figures 3A,B). These findings indicate that the depletion of TNIK in ECs does not adversely affect the uptake of Ad-GFP.

Next, we investigated the impact of TNIK depletion on the response of ECs to Ad-WT infection. HUVECs were initially transfected with either siCTRL or siTNIK. After a 48-hour incubation, these cells were transduced with Ad-WT at an MOI of 60. We closely monitored the development of CPE through daily microscopic examination (Figure 3C). Cells exhibiting CPE were identified based on three primary morphological alterations, which include (1) Changes in cell morphology, resulting in a round shape; (2) Fusion with adjacent cells to form syncytia; and (3) The presence of cytoplasmic/nuclear vacuoles due to the accumulation of viral components (31–35). The percentage of cells exhibiting CPE after Ad-WT transduction was quantified by calculating the ratio of cells with CPE to the total cell count. As illustrated in Figures 3D,E, the percentage of cells with CPE was higher in siTNIK-transfected HUVECs compared to siCTRL-transfected HUVECs. This observation indicates that TNIK depletion compromises the ability of ECs to defend against virus infection.

The plaque assay is a commonly used method for quantifying infectious particles (36). To determine the quantity of infectious virus particles within these cells, three freeze-thaw cycles were performed 72 h after Ad-WT transduction. Subsequently, a plaque-based assay using an immunohistochemical approach (36) was conducted to measure the number of infectious viral particles within HEK293 cell lysates. The results demonstrated an increase in the number of infectious virus particles in siTNIK-transfected HUVECs compared to siCTRL-transfected HUVECs (Figure 3F), consistent with the observations made during CPE cell counting (Figure 3E).

### 3.4 Impact of virus infection on TNIK and STAT expression

**Figure 4:**
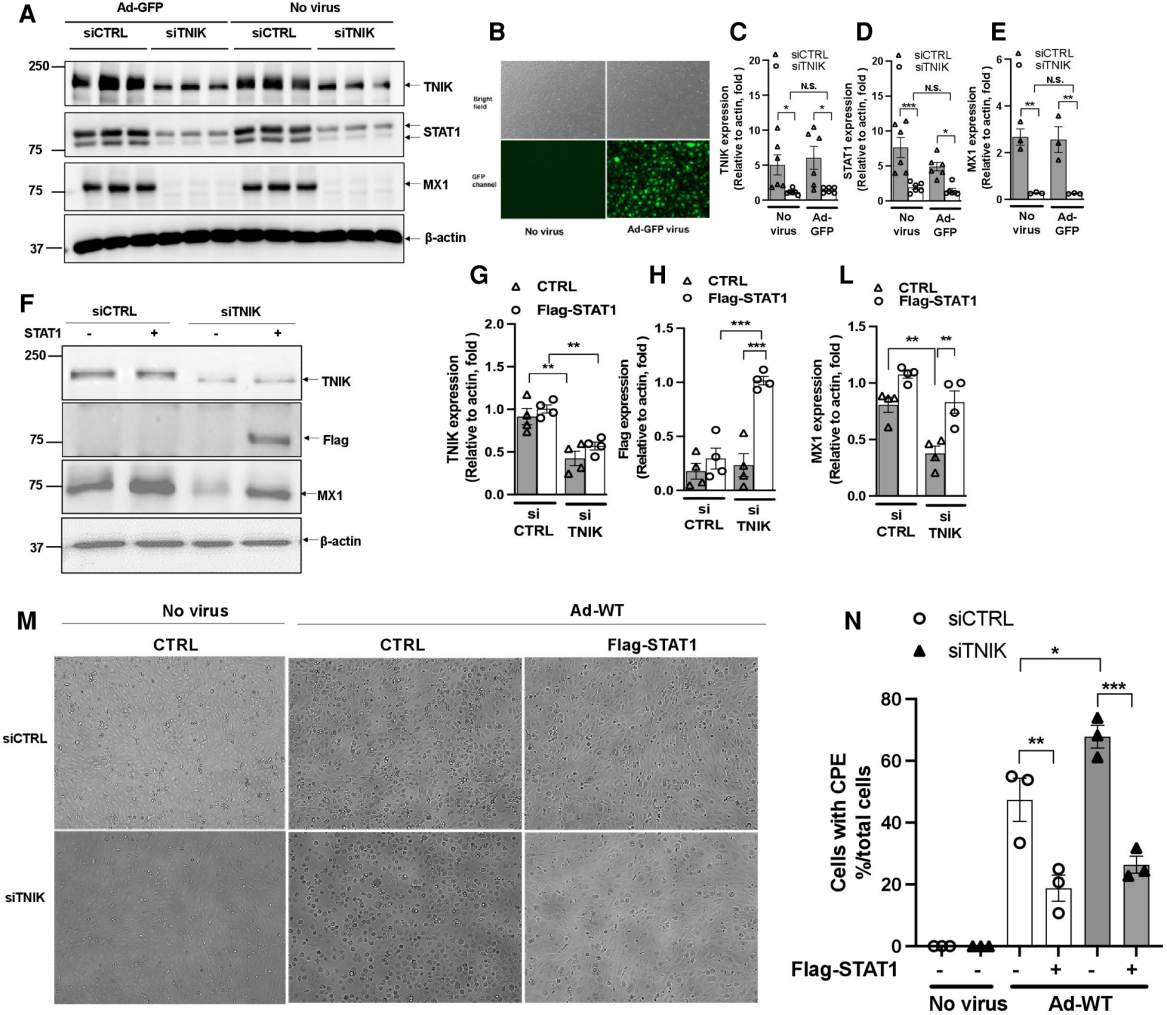
Effects of virus infection and STAT1 overexpression on TNIK in ECs. (**A,B**) HUVECs were initially transfected with siCTRL or siTNIK (50 nM). After a 48-hour incubation, these cells were transduced with Ad-GFP (MOI 60). Successful virus transduction was confirmed by the presence of GFP expression (**B**) Cell lysates were prepared using RIPA buffer, and Western blotting was performed to assess the expression levels of TNIK, STAT1, MX1, and actin (used as a loading control) (**A**) (**C,D,E**) The graphs represent the fold change in TNIK, STAT1, and MX1 expression relative to actin (used as a control) due to TNIK depletion, with or without Ad-GFP transduction. Mean ± SEM (*n* = 6, * indicates *p* < 0.05). (**F**) HUVECs were initially transfected with siCTRL or siTNIK (50 nM). After a 24-hour incubation, these cells were transfected with either pCMV-STAT1 or the parental pCMV-tag2B plasmid as a control. Following 24 h of plasmid transfection, total protein was extracted using 2× Laemmli buffer. Western blotting was then performed to assess the expression levels of TNIK, Flag (STAT1), and MX1. (**F**) shows representative immunoblot images displaying TNIK, Flag (STAT1), and MX1 expression. (**G,H,L**) The graphs illustrate the fold change in TNIK, STAT1, and MX1 expression relative to actin (used as a control) due to TNIK depletion. Mean ± SEM (*n* = 4, * indicates *p* < 0.05). (**M**) HUVECs were initially transfected with siCTRL or siTNIK (50 nM). After a 24-hour incubation, these cells were transfected with either pCMV-STAT1 or the parental pCMV-tag2B plasmid as a control. Following an additional 24 h after plasmid transfection, these cells were transduced with Ad-WT (MOI 60) to assess cytopathic effects. Cytopathic effect was observed 72 h after Ad-WT transduction. (**N**) The percentage of cells exhibiting CPE after Ad-WT transduction was quantified by calculating the ratio of cells exhibiting CPE to the total cell count.

To understand how virus infection affects TNIK protein expression, we conducted experiments in HUVECs. Initially, cells were transfected with either siCTRL or siTNIK at a concentration of 50 nM. After a 48-hour incubation, these cells were transduced with Ad-GFP at an MOI of 60, while others were left untransduced. Successful virus transduction was confirmed by observing GFP expression (Figure 4B). Subsequently, cell lysates were prepared using RIPA buffer, and Western blotting was performed to assess the expression levels of TNIK, STAT1, MX1, and actin as a loading control (Figure 4A). Our results, as shown in Figures 4A,D,E, indicate that TNIK depletion significantly reduced the expression levels of both STAT1 and MX1, regardless of whether virus transduction occurred or not. Importantly, virus transduction had no noticeable effect on TNIK expression (Figures 4A,C).

To assess the potential of STAT expression to counteract the effects of TNIK depletion, we conducted the following experiment. HUVECs were initially transfected with either siCTRL or siTNIK at a concentration of 50 nM. After a 24-hour incubation, these cells were further transfected with either pCMV-STAT1 or the parental pCMV-tag2B plasmid as a control. Following 24 h of plasmid transfection, total protein was extracted using 2× Laemmli buffer, and Western blotting was performed to evaluate the expression levels of TNIK, Flag (STAT1), and MX1, an IFN-induced protein with a crucial role in innate antiviral defense mechanisms. As shown in Figures 4F–L, TNIK depletion significantly reduced MX1 protein expression, and the overexpression of Flag-STAT1 effectively reversed this effect. It is worth noting that the extent of reversal by STAT1 overexpression was slightly attenuated in TNIK-depleted cells, although this difference did not reach statistical significance (Figures 4F–L). These findings suggest the presence of potential mechanisms, independent of TNIK-regulated STAT1/2 expression, which should be explored in future studies.

Additionally, some cells were transduced with Ad-WT at an MOI of 60 to assess CPE. The observation of the CPE induced by Ad-WT was performed under a microscope 72 h after Ad-WT transduction. As shown in Figures 4M,N, cells exhibiting CPE were observed in both siCTRL- and siTNIK-transfected groups, possibly due to the sequential transfection of siRNA and plasmid. However, fewer cells with STAT1 overexpression exhibited CPE compared to the control. This observation indicates that STAT1 overexpression inhibits cytopathic effects.

## 4 Discussion

Viruses have evolved mechanisms to commandeer host cells, co-opting their machinery to replicate and evade host immune responses. The interaction networks between viruses and host factors are pivotal for successful virus infection (43). Viruses employ various strategies for entering host cells, such as exploiting host endocytic mechanisms, although the precise mechanisms remain incompletely understood. Some viruses also utilize secretory pathways that involve the endoplasmic reticulum (ER) and Golgi apparatus (44, 45). Notably, the secretion, processing, and assembly of collagen are integral to numerous critical biological functions, and any disruptions in these processes can contribute to various pathologies. Collagen secretion entails the transportation of procollagen from the ER to the Golgi, a process known as ER-to-Golgi (anterograde) transport (44). Recent research has proposed that anterograde transport also plays a role in viral entry, replication, and assembly (46, 47). Collagen I, which plays a vital role in the formation of bone, skin, and tendon formation, relies on efficient secretion facilitated by the ER-to-Golgi transport of procollagen I. Research has demonstrated that the deletion or depletion of TNIK disrupts the secretion of procollagen I without affecting intracellular procollagen I protein levels (48). This disruption suggests that TNIK may be involved in viral entry and replication, particularly since some viruses exploit host secretory pathways, including the ER-to-Golgi transport. In fact, functional proteomics has identified TNIK as an interactive factor with the latent membrane protein 1 signalosome in primary human B-cells infected with EBV.

Our RNA-seq analysis uncovered a significant downregulation in the IFN pathway, chemokine, and cytokine responses upon TNIK depletion. This correlated with reduced protein expression of STAT1 and STAT2, the key transcription factors controlling IFN Type I and II responses. Dimerization and nuclear transport of STAT proteins are crucial for the regulation of downstream IFN-stimulated genes. Our gene dataset applied to the IFN gene set molecules from the GSEA database revealed the downregulation of 21 out of 29 genes in the IFN pathway, indicating that TNIK may indeed regulate IFN signaling. Our qRT-PCR analysis supported this, showing the downregulation of most IFN signaling-related genes, except for *irf9*, a known negative regulator of STATs. Additionally, cytokines and chemokines in the conditioned medium from siTNIK-transfected HUVECs, crucial for viral responses, showed significant decreases in CXCL10, CXCL11, and CCL5 compared to siCTRL-transfected HUVECs. These combined results strongly support that TNIK plays a pivotal role in regulating IFN signaling by modulating IFN transcription factors, specifically STAT1 and STAT2. Immunoblotting of lysates from siTNIK-transfected HUVECs further confirmed decreased STAT1 and STAT2 protein expression. While the involvement of TNIK in the c-Jun pathway and cytoskeleton regulation has been reported, the precise mechanism remains unclear (1). Subsequent research has primarily concentrated on its role in enhancing canonical Wnt signaling and promoting cancer cell proliferation (5, 49). Several studies have explored the potential of pharmacological TNIK inhibition to hinder cancer growth (4, 11, 50). However, prior to our study, no investigations had explored the role of TNIK in innate IFN pathways. Our research is the first to establish that TNIK indeed participates in the activation of IFN signaling in ECs.

Our results indicate a more significant exogenous STAT overexpression in siTNIK-transfected cells compared to siCTRL-transfected cells (Figures 4F,H). Zuo, Y. et al. (51) showed that endogenous STAT1 undergoes linear ubiquitination in cells, a process targeted by specific enzymes for degradation. We propose that in siCTRL-transfected cells, both exogenously expressed and endogenously expressed STAT1 may be subjected to enzyme-mediated degradation, primarily affecting the exogenously expressed STAT1 due to its posttranslational status, leading to rapid turnover. Conversely, the lower levels of endogenous STAT1 in siTNIK-transfected cells may result in decreased enzymatic activity, resulting in increased exogenous STAT1 stability. Further experiments in future studies are necessary to validate this possibility.

**Figure 5:**
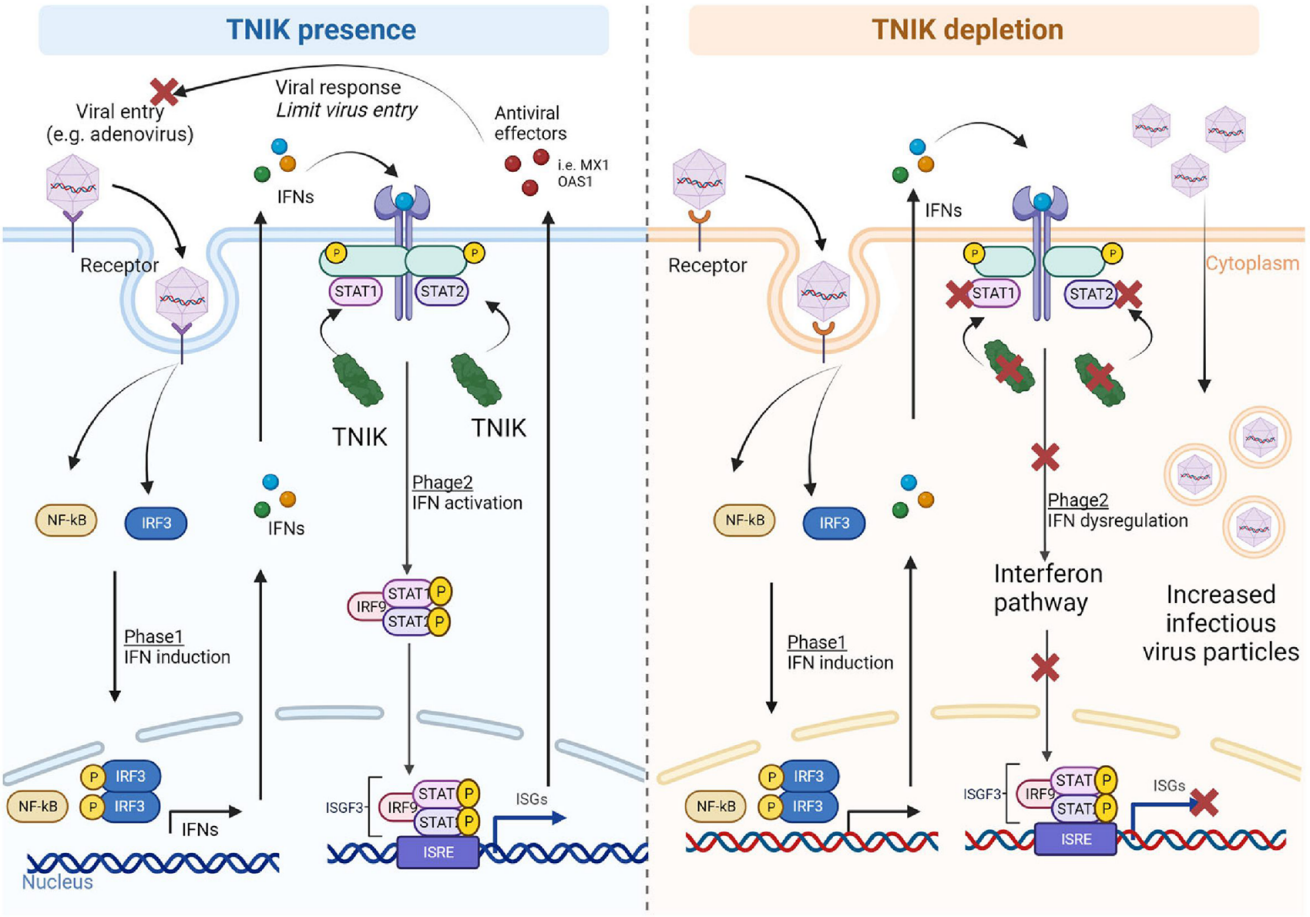
Proposed mechanism. In the presence of TNIK, virus particles entering the cells activate two phases of the IFN system: (1) IFN induction, upregulating IFN-related genes through NF-*κ*B and IRF gene regulators, and (2) IFN action, where secreted IFNs during IFN induction phase activate the IFN signaling pathway with STAT playing critical roles. TNIK is proposed to regulate STAT. Through the IFN system, various antiviral effectors are secreted to counteract viral entry and responses. In the absence of TNIK, the IFN action phase is deactivated due to STAT depletion resulting from TNIK depletion, leading to a lack of antiviral effector production and allowing more virus entry into the cells.

We propose several potential mechanisms by which TNIK may exert this regulatory role. These mechanisms could include direct or indirect transcriptional control of STATs, stabilization of STATs to prevent their degradation, or acting as a binding or complex-initiating factor essential for the transcriptional activity of STATs. Consequently, these mechanisms lead to the downstream downregulation of multiple IFN genes (Figure 5). Our future studies will work towards elucidating and confirming these mechanisms. Additionally, in a gene expression study involving patients with Kawasaki disease, a systemic vasculitis syndrome of unknown etiology, TNIK was identified as one of the major dysregulated genes (52). Thus, our findings regarding the role of TNIK in IFN regulation may hold crucial implications for understanding and managing systemic inflammatory diseases that significantly affect the vasculatures, such as Lupus Erythematosus and Kawasaki disease.

In our study, we opted to use adenoviral infection because adenovirus has the capability to trigger IFN responses (53). The adenoviral life cycle encompasses various stages, including attachment, internalization, replication, biosynthesis, assembly, and release of viral progenies (54, 55). Adenovirus enters host cells through receptor-mediated endocytosis involving the Coxsackievirus and adenovirus receptor (CAR). It's worth noting that some cells lacking CAR expression may require a higher viral infectious unit per cell (ranging from 5 × 10^3^ to 7.5 × 10^3^) to achieve a 90% transduction efficiency, even when standard transfection reagents are used (56). ECs typically have a low level of CAR expression (57). While there is not a well-established average rate of adenoviral infection in HUVECs, adenovirus follows a Poisson distribution (f(x) = (*λ*χ/χ!)e-*λ*, where (f(x) represents the probability, *λ* is the average rate, and χ is the variable) rather than a linear distribution (53). Considering this distribution, we anticipate that an MOI of 30–60, equivalent to roughly 4–8 adenoviral particles per HUVEC, should suffice for standard viral transduction.

Upon entering host cells, adenovirus initiates the production of early mRNAs, resulting in the synthesis of early viral regulatory proteins. In the later stages of the adenoviral life cycle, structural proteins are generated, leading to the assembly and release of fully mature viruses through cell lysis, a process distinct from that of most other viruses. Notably, adenoviral proteins have evolved to counteract IFN (58), which could potentially complicate our study. For our research, we deliberately chose to use a wild-type adenovirus instead of its replication-defective counterpart. The rationale behind this choice is that wild-type viruses, in contrast to their replication-defective counterparts, have the ability to replicate outside of cells engineered to deliver the E3 protein in trans (54). These wild-type viruses can hijack protein synthesis machinery of the host cells to replicate themselves, ultimately leading to cell lysis. It is important to mention that other studies have confirmed the expression of CARs in ECs (57, 59). Therefore, it is expected that adenovirus enters these cells through receptor-mediated endocytosis mechanisms.

In both siCTRL-transfected HUVECs and HUVECs without siRNA transfection, no CPE or cell lysis was observed even on the fourth day after virus transduction. However, in siTNIK-transfected HUVECs, we observed CPE and the presence of intercellular vacuoles by the fourth day, indicating that the depletion of TNIK compromises the protective and pro-survival mechanisms (Figures 3D–F). Interestingly, this phenotype was not detected in siTNIK-transfected HUVECs without Ad-WT transduction, where ECs displayed the classic cytopathic morphology associated with viral infection. These findings strongly suggest that TNIK plays a role in safeguarding ECs from adenovirus-induced cell death, likely through the upregulation of the IFN pathway. Additionally, our data revealed that the number of infectious viral particles was higher in siTNIK-transfected HUVECs. This observation indicates that knocking down TNIK results in the downregulation of IFN activity, thus compromising the ability of cells to defend against adenoviral replication (Figures 3D–F).

ECs play a pivotal role in maintaining vascular tone and homeostasis, and their activation is closely linked to the development of various pathologies, including vasculitis and atherosclerosis (45, 60–61). ECs are equipped with receptors for both IFN type I and II, as well as cytokines and chemokines produced by activated immune cells and cells infected by viruses (62). ECs are responsive to viral infections, and the evidence of viral infection in cultured ECs, such as cytopathology, viral growth curves, and antigen detection, highlights their role in initiating vessel wall injury. These cells are considered to serve as supportive innate immune cells capable of suppressing virus replication by initiating an autologous immune response (26, 63). Nevertheless, the virus can persist in ECs, continuing to replicate at a slow rate, even after the initial infection has been eliminated. While our study primarily focused on adenovirus, existing literature suggests that influenza virus infection in ECs may similarly persist without causing cell lysis (45, 64, 65). Our findings emphasize the concept that viruses can endure within ECs by subduing their replication, without complete elimination of the virus. This implies that ECs may harbor viral infections over the long term, potentially leading to alterations in these cells that contribute to the earlier onset of cardiovascular diseases like atherosclerosis. Therefore, our research reveals a novel mechanism by which viruses can establish persistent infections in ECs, highlighting the potential long-lasting impacts on cardiovascular health.

Various human viruses have been linked to an increased risk of atherosclerosis and cardiovascular disease (45, 66, 67). Large-scale studies have shown a significant correlation between influenza infection and cardiac issues, including an exacerbation of atherogenesis (68–70). Our data suggest that potential virus infection of ECs may lead to prolonged IFN signaling, a factor linked to atherogenic plaque development (71). Consequently, our findings propose that persistent EC infections may have enduring implications for cardiovascular diseases, potentially offering insight into how a seemingly eradicated initial viral infection can continue undetected. Further *in vivo* research is required to ascertain whether this persistent inflammatory response might induce epigenetic changes in ECs, leading to their activation, macrophage recruitment, prothrombotic events, and the eventual formation of atherogenic plaques (72, 73). Through the significant reduction of TNIK expression via siRNA knockdown, we observed a corresponding delay in the lysis of ECs induced by adenovirus in siTNIK-transfected cells. This delayed lysis, as opposed to immediate cell ablation, likely accounts for the extended timeframe required for cell lysis in samples with reduced TNIK expression. Nevertheless, the specific duration for adenovirus to induce EC lysis *in vivo* remains uncertain, highlighting the importance of conducting further animal studies to address this critical question.

## Data Availability

For any additional data, analytic methods, or study materials that are essential for supporting the findings of this study, please feel free to request them from the corresponding author, and they will be made available upon reasonable request.

## Supplementary material

Supplementary Figure S1 IPA analysis reveals the role of TNIK in EC inflammatory response and infectious diseases. (**A**) Heatmap displaying DEGs in ECs following TNIK depletion: 312 upregulated DEGs and 355 downregulated DEGs (log2 fold change >1, *p*-value <0.05). (**B**) IPA-based heatmaps of functional hierarchy showing the analysis of diseases and functions associated with differential gene sets expressed in siTNIK-transfected HAECs, including significance and predicted activity states. Color-coded scales indicate function change directions determined by activation *z*-score value, where “Orange” indicates upregulation, “Blue” indicates downregulation, “White” indicates a *z*-score of 0, and “Gray” indicates data not available. The size of each box reflects the *p*-value (the larger the box, the smaller the *p*-value). (**C**) The squares represent changes in diseases/functions predicted in siTNIK-transfected HAECs. The “Inflammatory responses” and “Infectious diseases” categories have high significance, with TNIK depletion downregulating inflammatory responses (*Z*-score range −3.518 to −1.112) and upregulating the potential for infectious diseases (*Z*-score range 3.011–3.801).

Supplementary Figure S2 GFP Expression and Transduction Efficiency of ECs at Different MOIs Following Ad-GFP Virus Transduction. HUVECs were transduced with Ad-GFP at various MOIs, including MOI 0, MOI 60, MOI 120, MOI 240, MOI 360, and MOI 475. GFP virus expression was visualized under a fluorescent microscope 24 h post-transduction (scale bar: 20 µm).
