# Supplementary material for: TNIK regulation of interferon signaling and endothelial cell response to virus infection
Source: Front Cardiovasc Med. 2024 Jan 9;10:1213428. doi: 10.3389/fcvm.2023.1213428 (PMC10803426; doi:10.3389/fcvm.2023.1213428)
Supplement: Supplementary file 1 [file Table1.docx]

**Supplementary Table 1**. List of qRT-PCRT primers.

| **Gene name** | **Forward (5’-3’)** | **Reverse (5’-3’)** |
| --- | --- | --- |
| IFIT1 | GCCTTGCTGAAGTGTGGAGGAA | ATCCAGGCGATAGGCAGAGATC |
| IFIT2 | GGAGCAGATTCTGAGGCTTTGC | GGATGAGGCTTCCAGACTCCAA |
| IFIT3 | CCTGGAATGCTTACGGCAAGCT | GAGCATCTGAGAGTCTGCCCAA |
| IFI6 | TGATGAGCTGGTCTGCGATCCT | GTAGCCCATCAGGGCACCAATA |
| IFI30 | GTGGGAGTTCAAGTGCCAGCAT | GCAGACAATGGTCAGGAAGGCT |
| IFI35 | CACGATCAACATGGAGGAGTGC | GGCAGGAAATCCAGTGACCAAC |
| MX1 | GGCTGTTTACCAGACTCCGACA | CACAAAGCCTGGCAGCTCTCTA |
| OAS1 | AGGAAAGGTGCTTCCGAGGTAG | GGACTGAGGAAGACAACCAGGT |
| IRF9 | CCACCGAAGTTCCAGGTAACAC | AGTCTGCTCCAGCAAGTATCGG |
| ISG20 | ACACGTCCACTGACAGGCTGTT | ATCTTCCACCGAGCTGTGTCCA |
| RSAD2 | CCAGTGCAACTACAAATGCGGC | CGGTCTTGAAGAAATGGCTCTCC |
| IFIH1 | GCTGAAGTAGGAGTCAAAGCCC | CCACTGTGGTAGCGATAAGCAG |
| RTP4 | GACGCTGAAGTTGGATGGCAAC | GTGGCACAGAATCTGCACTTGG |
| CXCL10 | GGTGAGAAGAGATGTCTGAATCC | GTCCATCCTTGGAAGCACTGCA |
| CXCL11 | AAGGACAACGATGCCTAAATCCC | CAGATGCCCTTTTCCAGGACTTC |
| CCL5 | CCTGCTGCTTTGCCTACATTGC | ACACACTTGGCGGTTCTTTCGG |
