# Supplementary material for: TNIK regulation of interferon signaling and endothelial cell response to virus infection
Source: Front Cardiovasc Med. 2024 Jan 9;10:1213428. doi: 10.3389/fcvm.2023.1213428 (PMC10803426; doi:10.3389/fcvm.2023.1213428)
Supplement: Supplementary file 2 [file Table2.docx]

**Supplementary Table 2:** **Predicted downregulation of genes related to ‘interferon pathway’ and ‘Role of Hypercytokinemia/ Hyperchemokinemia in the pathogenesis of influenza” in siTNIK-transfected HAECs vs siCTRL-transfected HAECs.**

| **Symbol** | **Entrez Gene Name** | **Gene in dataset** | **Expr Log Ratio** |
| --- | --- | --- | --- |
| *Interferon signaling pathway* | | | |
| IFI6 | interferon alpha inducible protein 6 | IFI6 | -2.464 |
| IFI35 | interferon induced protein 35 | IFI35 | -2.444 |
| IFIT1 | interferon induced protein with tetratricopeptide repeats 1 | IFIT1 | -3.414 |
| IFIT3 | interferon induced protein with tetratricopeptide repeats 3 | IFIT3 | -3.627 |
| IFITM1 | interferon induced transmembrane protein 1 | IFITM1 | -3.338 |
| IFITM3 | interferon induced transmembrane protein 3 | IFITM3 | -1.333 |
| ISG15 | ISG15 ubiquitin like modifier | ISG15 | -2.61 |
| MX1 | MX dynamin like GTPase 1 | MX1 | -2.631 |
| OAS1 | 2'-5'-oligoadenylate synthetase 1 | OAS1 | -2.726 |
| PSMB8 | proteasome 20S subunit beta 8 | PSMB8 | -1.507 |
| STAT1 | signal transducer and activator of transcription 1 | STAT1 | -1.545 |
| STAT2 | signal transducer and activator of transcription 2 | STAT2 | -1.285 |
| TAP1 | transporter 1, ATP binding cassette subfamily B member | TAP1 | -2.028 |
| *Role of Hypercytokinemia/ Hyperchemokinemia in the pathogenesis of Influenza* | | | |
| CASP1 | caspase 1 | CASP1 | -1.266 |
| CCL5 | C-C motif chemokine ligand 5 | CCL5 | -5.28 |
| CXCL10 | C-X-C motif chemokine ligand 10 | CXCL10 | -3.228 |
| DDX58 | DExDH-box helicase 58 | DDX58 | -2.256 |
| EIF2AK2 | eukaryotic translation initiation factor 2 alpha kinase 2 | EIF2AK2 | -1.322 |
| IFIT2 | interferon induced protein with tetratricopeptide repeats 2 | IFIT2 | -3.534 |
| IFIT3 | interferon induced protein with tetratricopeptide repeats 3 | IFIT3 | -3.627 |
| IL1A | interleukin 1 alpha | IL1A | -1.242 |
| IRF7 | interferon regulatory factor 7 | IRF7 | -2.191 |
| ISG15 | ISG15 ubiquitin like modifier | ISG15 | -2.61 |
| ISG20 | interferon stimulated exonuclease gene 20 | ISG20 | -2.756 |
| OAS2 | 2'-5'-oligoadenylate synthetase 2 | OAS2 | -2.359 |
| OAS3 | 2'-5'-oligoadenylate synthetase 3 | OAS3 | -2.281 |
| RSAD2 | radical S-adenosyl methionine domain containing 2 | RSAD2 | -3.629 |
| TLR3 | toll like receptor 3 | TLR3 | -2.276 |
