# Supplementary material for: TNIK regulation of interferon signaling and endothelial cell response to virus infection
Source: Front Cardiovasc Med. 2024 Jan 9;10:1213428. doi: 10.3389/fcvm.2023.1213428 (PMC10803426; doi:10.3389/fcvm.2023.1213428)
Supplement: Supplementary file 3 [file Table3.docx]

**Supplementary Table 3: Predicted upregulation of genes relating to infectious disease category in siTNIK-transfected HAEC.**

|  |  |  |  |  |  |
| --- | --- | --- | --- | --- | --- |
| **Categories** | **Diseases or Functions Annotation** | **p-value** | **Predicted Activation State** | **Activation z-score** | **# Molecules** |
| **Infectious Diseases** | **Viral Infection** | **3.62E-27** | **Increased** | **3.452** | **110** |
| **Infectious Diseases** | **Replication of virus** | **4.1E-24** | **Increased** | **3.801** | **55** |
| Infectious Diseases | Replication of RNA virus | 2.15E-21 | Increased | 3.633 | 49 |
| Infectious Diseases | Replication of vesicular stomatitis virus | 1.31E-13 | Increased | 2.633 | 15 |
| Infectious Diseases | Infection of mammalian | 2.98E-12 | Increased | 3.353 | 28 |
| Infectious Diseases | Replication of Flaviviridae | 4.86E-11 | Increased | 3.123 | 14 |
| Infectious Diseases | Replication of Murine herpesvirus 4 | 8.33E-10 | Increased | 2.646 | 7 |
| Infectious Diseases | Replication of Hepatitis C virus | 1.5E-09 | Increased | 2.814 | 12 |
| Infectious Diseases | Replication of murine hepatitis virus | 6.92E-09 | Increased | 2.008 | 7 |
| Infectious Diseases | Infection by Orthomyxoviridae | 2.77E-08 | Increased | 2.438 | 9 |
| Infectious Diseases | Replication of coronavirus | 0.000000151 | Increased | 2.575 | 10 |
| Infectious Diseases | Infection by Picornaviridae | 0.00000344 | Increased | 2.411 | 7 |
| **Infectious Diseases** | **Production of virus** | **0.00000929** | **Increased** | **3.011** | **10** |
