# Supplementary material for: TNIK regulation of interferon signaling and endothelial cell response to virus infection
Source: Front Cardiovasc Med. 2024 Jan 9;10:1213428. doi: 10.3389/fcvm.2023.1213428 (PMC10803426; doi:10.3389/fcvm.2023.1213428)
Supplement: Supplementary file 4 [file Table4.docx]

**Supplementary Table 4:** **Predicted downregulation of genes related to inflammatory response category in siTNIK-transfected HAEC.**

| **Categories** | **Diseases or Functions Annotation** | **p-value** | **Predicted Activation State** | **Activation z-score** | **# Molecules** |
| --- | --- | --- | --- | --- | --- |
| **Antimicrobial Response, Inflammatory Response** | **Antiviral response** | **8.38E-38** | **Decreased** | **-3.518** | **46** |
| **Antimicrobial Response, Inflammatory Response** | **Antimicrobial response** | **2.09E-28** | **Decreased** | **-3.518** | **47** |
| **Inflammatory Response** | **Innate immune response** | **5.87E-18** | **Decreased** | **-1.112** | **30** |
| Inflammatory Response | Inflammation of body cavity | 3.08E-11 | Decreased | -2.231 | 56 |
| Inflammatory Response | Immune response of cells | 6.87E-11 | Decreased | -2.727 | 39 |
| Cellular Movement, Hematological System Development and Function, Immune Cell Trafficking, Inflammatory Response | Cell movement of macrophages | 1.11E-08 | Decreased | -2.758 | 23 |
| Cellular Movement, Hematological System Development and Function, Immune Cell Trafficking, Inflammatory Response | Cellular infiltration by phagocytes | 2.02E-07 | Decreased | -2.411 | 21 |
| Cell-To-Cell Signaling and Interaction, Inflammatory Response | Response of dendritic cells | 5.51E-07 | Decreased | -2.236 | 9 |
| Antimicrobial Response, Inflammatory Response | Antiviral response of cells | 2.6E-06 | Decreased | -2.63 | 7 |
| Cellular Movement, Hematological System Development and Function, Immune Cell Trafficking, Inflammatory Response | Cell movement of neutrophils | 1.02E-05 | Decreased | -2.531 | 19 |
