# Supplementary material for: TNIK regulation of interferon signaling and endothelial cell response to virus infection
Source: Front Cardiovasc Med. 2024 Jan 9;10:1213428. doi: 10.3389/fcvm.2023.1213428 (PMC10803426; doi:10.3389/fcvm.2023.1213428)
Supplement: Supplementary file 5 [file Table5.docx]

**Supplementary Table 5: predicted downregulation of genes related to immune cell trafficking category in siTNIK-transfected HAEC.**

| **Categories** | **Diseases or Functions Annotation** | **p-value** | **Predicted Activation State** | **Activation z-score** | **# Molecules** |
| --- | --- | --- | --- | --- | --- |
| Cellular Movement, Hematological System Development and Function, Immune Cell Trafficking | Cellular infiltration by leukocytes | 7.53E-09 | Decreased | -2.319 | 29 |
| Cellular Movement, Hematological System Development and Function, Immune Cell Trafficking, Inflammatory Response | Cell movement of macrophages | 1.11E-08 | Decreased | -2.758 | 23 |
| **Cellular Movement, Hematological System Development and Function, Immune Cell Trafficking, Inflammatory Response** | **Cellular infiltration by phagocytes** | **2.02E-07** | **Decreased** | **-2.411** | **21** |
| Cell-To-Cell Signaling and Interaction, Cellular Movement, Hematological System Development and Function, Immune Cell Trafficking | Recruitment of leukocytes | 5.78E-07 | Decreased | -2.289 | 21 |
| Cellular Movement, Hematological System Development and Function, Immune Cell Trafficking | Cell movement of granulocytes | 4.19E-06 | Decreased | -2.392 | 22 |
| Cellular Movement, Hematological System Development and Function, Immune Cell Trafficking, Inflammatory Response | Cell movement of neutrophils | 1.02E-05 | Decreased | -2.531 | 19 |
