# Supplementary material for: TNIK regulation of interferon signaling and endothelial cell response to virus infection
Source: Front Cardiovasc Med. 2024 Jan 9;10:1213428. doi: 10.3389/fcvm.2023.1213428 (PMC10803426; doi:10.3389/fcvm.2023.1213428)
Supplement: Supplementary file 8 [file Table8.docx]

**Supplementary Table 8. Predicted upregulation of genes related to Hematological Disease category in siTNIK-transfected HAEC.**

| **Categories** | **Diseases or Functions Annotation** | **p-value** | **Predicted Activation State** | **Activation z-score** | **# Molecules** |
| --- | --- | --- | --- | --- | --- |
| Cancer, Hematological Disease, Organismal Injury and Abnormalities | Hematologic cancer of cells | 4.08E-11 | Increased | 2.064 | 88 |
| Cancer, Hematological Disease, Immunological Disease, Organismal Injury and Abnormalities | Lymphocytic cancer | 8.21E-11 | Increased | 2.064 | 94 |
| **Cancer, Hematological Disease, Organismal Injury and Abnormalities** | **Lymphocytic neoplasm** | **8.81E-11** | **Increased** | **2.064** | **94** |
| Cancer, Hematological Disease, Organismal Injury and Abnormalities | Hematologic cancer | 7.42E-09 | Increased | 2.264 | 112 |
