# Supplementary material for: TNIK regulation of interferon signaling and endothelial cell response to virus infection
Source: Front Cardiovasc Med. 2024 Jan 9;10:1213428. doi: 10.3389/fcvm.2023.1213428 (PMC10803426; doi:10.3389/fcvm.2023.1213428)
Supplement: Supplementary file 9 [file Table9.docx]

**Supplementary Table 9. Predicted upregulation of genes related to Cancers category in siTNIK-transfected HAEC.**

| **Categories** | **Diseases or Functions Annotation** | **p-value** | **Predicted Activation State** | **Activation z-score** | **# Molecules** |
| --- | --- | --- | --- | --- | --- |
| **Cancer, Organismal Injury and Abnormalities** | **Neoplasia of cells** | **5.19E-14** | **Increased** | **2.121** | **181** |
| Cancer, Hematological Disease, Organismal Injury and Abnormalities | Hematologic cancer of cells | 4.08E-11 | Increased | 2.064 | 88 |
| Cancer, Hematological Disease, Immunological Disease, Organismal Injury and Abnormalities | Lymphocytic cancer | 8.21E-11 | Increased | 2.064 | 94 |
| Cancer, Hematological Disease, Organismal Injury and Abnormalities | Lymphocytic neoplasm | 8.81E-11 | Increased | 2.064 | 94 |
| Cancer, Organismal Injury and Abnormalities | Lymphoreticular neoplasm | 2.8E-09 | Increased | 2.348 | 96 |
| Cancer, Hematological Disease, Organismal Injury and Abnormalities | Hematologic cancer | 7.42E-09 | Increased | 2.264 | 112 |
| Cancer, Organismal Injury and Abnormalities | Neoplasia of tumor cell lines | 8.51E-06 | Increased | 2.139 | 25 |
