# Supplementary figures and images for: TNIK regulation of interferon signaling and endothelial cell response to virus infection
Source: Front Cardiovasc Med. 2024 Jan 9;10:1213428. doi: 10.3389/fcvm.2023.1213428 (PMC10803426; doi:10.3389/fcvm.2023.1213428)

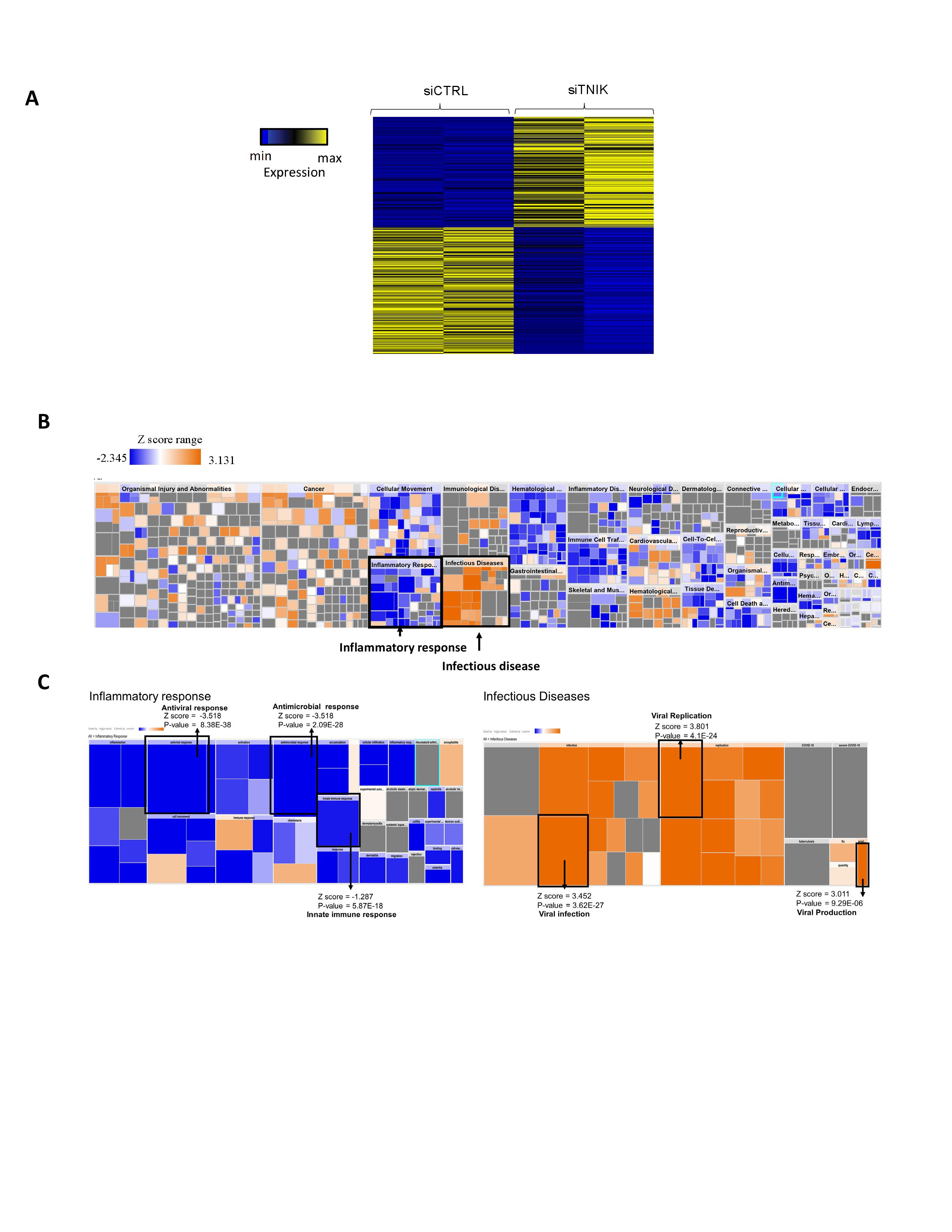

Supplement: Supplementary Figure S1 — IPA analysis reveals the role of TNIK in EC inflammatory response and infectious diseases. (A) Heatmap displaying DEGs in ECs following TNIK depletion: 312 upregulated DEGs and 355 downregulated DEGs (log2 fold change >1, p-value <0.05). (B) IPA-based heatmaps of functional hierarchy showing the analysis of diseases and functions associated with differential gene sets expressed in siTNIK-transfected HAECs, including significance and predicted activity states. Color-coded scales indicate function change directions determined by activation z-score value, where “Orange” indicates upregulation, “Blue” indicates downregulation, “White” indicates a z-score of 0, and “Gray” indicates data not available. The size of each box reflects the p-value (the larger the box, the smaller the p-value). (C) The squares represent changes in diseases/functions predicted in siTNIK-transfected HAECs. The “Inflammatory responses” and “Infectious diseases” categories have high significance, with TNIK depletion downregulating inflammatory responses (Z-score range −3.518 to −1.112) and upregulating the potential for infectious diseases (Z-score range 3.011–3.801). [file Image1.jpeg]

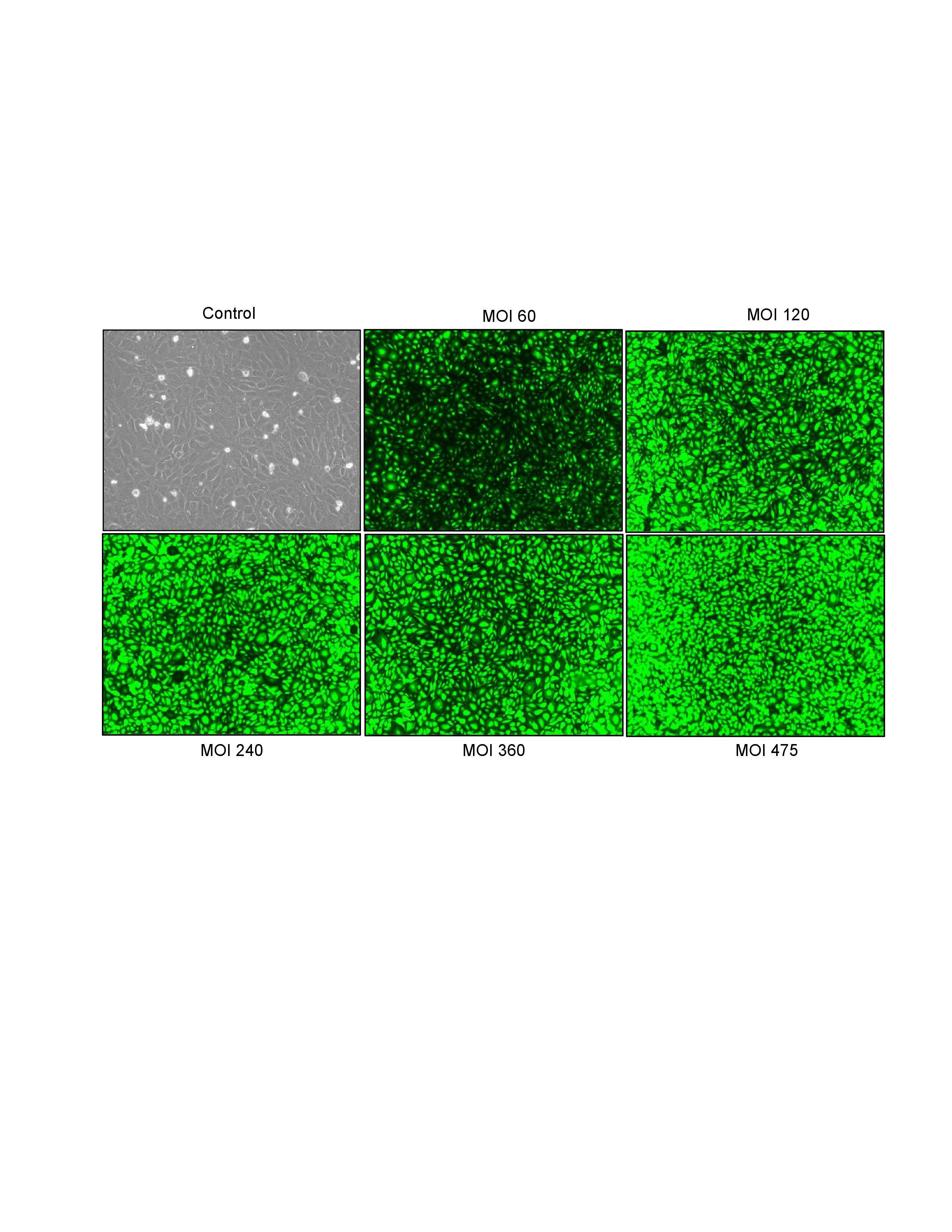

Supplement: Supplementary Figure S2 — GFP Expression and Transduction Efficiency of ECs at Different MOIs Following Ad-GFP Virus Transduction. HUVECs were transduced with Ad-GFP at various MOIs, including MOI 0, MOI 60, MOI 120, MOI 240, MOI 360, and MOI 475. GFP virus expression was visualized under a fluorescent microscope 24 h post-transduction (scale bar: 20 µm). [file Image2.jpeg]
